# Symbolic Analysis of the Heart Rate Variability During the Plateau Phase Following Maximal Sprint Exercise

**DOI:** 10.3389/fphys.2021.632883

**Published:** 2021-03-23

**Authors:** Jorge L. Storniolo, Beatrice Cairo, Alberto Porta, Paolo Cavallari

**Affiliations:** ^1^Department of Pathophysiology and Transplantation, Human Physiology Section, University of Milan, Milan, Italy; ^2^Department of Biomedical Sciences for Health, University of Milan, Milan, Italy; ^3^Department of Cardiothoracic, Vascular Anesthesia and Intensive Care, IRCCS Policlinico San Donato, Milan, Italy

**Keywords:** autonomic nervous system, autoregressive process, cardiac control, heart period, physical exercise, spectral analysis, sport physiology, surrogate data

## Abstract

Cardiac autonomic control is commonly assessed *via* the analysis of fluctuations of the temporal distance between two consecutive R-waves (RR). Cardiac regulation assessment following high intensity physical exercise is difficult due to RR non-stationarities. The very short epoch following maximal sprint exercise when RR remains close to its lowest value, i.e., the PLATEAU, provides the opportunity to evaluate cardiac regulation from stationary RR sequences. The aim of the study is to evaluate cardiac autonomic control during PLATEAU phase following 60-m maximal sprint and compare the results to those derived from sequences featuring the same length as the PLATEAU and derived from pre-exercise and post-exercise periods. These sequences were referred to as PRE and POST sequences. RR series were recorded in 21 subjects (age: 24.9 ± 5.1 years, 15 men and six women). We applied a symbolic approach due to its ability to deal with very short RR sequences. The symbolic approach classified patterns formed by three RRs according to the sign and number of RR variations. Symbolic markers were compared to more classical time and frequency domain indexes. Comparison was extended to simulated signals to explicitly evaluate the suitability of methods to deal with short variability series. A surrogate test was applied to check the null hypothesis of random fluctuations. Over simulated data symbolic analysis was able to separate dynamics with different spectral profiles provided that the frame length was longer than 10 cardiac beats. Over real data the surrogate test indicated the presence of determinism in PRE, PLATEAU, and POST sequences. We found that the rate of patterns with two variations with unlike sign increased during PLATEAU and in POST sequences and the frequency of patterns with no variations remained unchanged during PLATEAU and decreased in POST compared to PRE sequences. Results indicated a sustained sympathetic control along with an early vagal reactivation during PLATEAU and a shift of the sympathovagal balance toward vagal predominance in POST compared to PRE sequences. Time and frequency domains markers were less powerful because they were dominated by the dramatic decrease of RR variance during PLATEAU.

## Introduction

Heart rate variability (HRV), defined as the beat-to-beat variations of the time interval between two consecutive R-wave peaks (RR) detected over the electrocardiogram, has been historically processed to infer cardiac autonomic control in physiological and pathological conditions (Pomeranz et al., 1985; Task Force of the European Society of Cardiology the North American Society of Pacng Electrophysiology, 1996; Qiu et al., 2017). Among other applications, HRV allows the evaluation of the fitness status of a subject and provides predictive information about cardiac morbidity and mortality (Imai et al., 1994; Buchheit et al., 2007; Michael et al., 2017). The wide exploitation of HRV markers is owing to the possibility of gauging the balance between sympathetic and vagal controls *via* simple recording devices even outside the well-controlled conditions of the laboratory. Sympathovagal balance is usually estimated *via* short-term HRV analysis by assessing the power of low frequency (LF, from 0.04 to 0.15 Hz) and high frequency (HF, from 0.15 to 0.5 Hz) oscillations (Pomeranz et al., 1985; Task Force of the European Society of Cardiology the North American Society of Pacng Electrophysiology, 1996; Pagani et al., 1997; Shaffer and Ginsberg, 2017). In order to assure a reliable estimation of the amplitude of the LF and HF oscillations a typical rule of thumb suggests to take a series length longer than 5 times the slowest meaningful oscillation that the spectral analysis would like to resolve (i.e., 0.04 Hz). This requirement imposes that short-term frequency domain analysis of HRV is carried out over RR series longer than 125 s (i.e., longer than 125 RR values with a RR mean of 1 s). Shorter series are usually analyzed only in time domain because in this domain no specific HRV features were extracted and descriptive statistical quantities are the typical target (i.e., mean and variance).

Symbolic analysis has been proposed to assess cardiac autonomic control (Voss et al., 1996; Wessel et al., 2000; Porta et al., 2001, 2015, 2020; Baumert et al., 2002; Cysarz et al., 2012, 2013, 2015; Silva et al., 2017). This approach is based on the classification of very short RR patterns lasting four cardiac beats, namely three consecutive RRs, and the estimation of the rate of specific pattern families. This strategy allows the application of symbolic analysis over much shorter series than those utilized in short-term frequency domain analysis of HRV. More specifically, the rate of patterns with very small variations, i.e., stable patterns, was found to be correlated with the relevance of sympathetic control (Guzzetti et al., 2005; Porta et al., 2007b), while the frequency of high variable patterns was linked with an active vagal control and sympathetic withdrawal (Guzzetti et al., 2005; Porta et al., 2007a,b). The appropriateness of this methodology over very short time series makes it suitable in contexts, such as exercise physiology and sport medicine (Borresen and Lambert, 2008; Coote, 2010; Michael et al., 2017), in which HRV recordings cannot be extended due to the intrinsically short duration of epoch under scrutiny and/or loss of stationarity over longer periods.

The transition between the end of the exercise and recovery onset features an interesting, and relevant, state of the sympathovagal balance reflecting the persistence of a sizable sympathetic control in presence of a rapid parasympathetic reactivation (Imai et al., 1994; Martinmäki and Rusko, 2008). As a consequence of this particular state of the sympathovagal balance, RR variability recordings starting just after maximal exercise cessation might exhibit a plateau phase (PLATEAU) (Storniolo et al., 2020). This phase is usually discarded when the recovery after exercise is analyzed and beat-to-beat RR dynamic is fitted with exponential functions to quantify rapidity of the RR recovery (Imai et al., 1994; Buchheit et al., 2007; Peçanha et al., 2014). Conversely, it has been recently stressed that the PLATEAU observed after maximal sprint test contains relevant information about the individual characteristic of cardiac autonomic control because its duration is positively correlated with the pre-exercise state and post-exercise variation of the sympathovagal balance (Storniolo et al., 2020).

A remarkable feature of the PLATEAU is its limited duration (Storniolo et al., 2020). This characteristic makes spectral analysis to be an unsuitable tool for the description of sympathovagal interactions. Moreover, during this phase the interactions between the two different limbs of the autonomic nervous system are likely to be non-linear (Savin et al., 1982; Michael et al., 2017). This characteristic provides a further reason to avoid linear techniques like spectral analysis and to prefer symbolic analysis.

The aim of this study is to examine the beat-to-beat RR variability during PLATEAU observed just after a maximal sprint exercise *via* symbolic analysis. We hypothesize that during PLATEAU the high value of heart rate, the smallness of amplitude of the RR variations and the difficulties in analyzing RR dynamics during this period due to its shortness could mask the early vagal reactivation. If this study could confirm the robustness of symbolic analysis in analyzing very short data sequences following maximal exercise, the systematic application of this tool might open new possibilities of investigating the sympathovagal interactions from very short data sequences. We also analyze the RR variability before and after maximal sprint exercise over sequences of the same length as during PLATEAU. Results are compared with more traditional time and frequency domain techniques (Task Force of the European Society of Cardiology the North American Society of Pacng Electrophysiology, 1996) to further stress the potential of symbolic analysis. Simulated series were generated to better understand the ability of the methods over very short data sequences (Tobaldini et al., 2009) and a surrogate test was applied to check the null hypothesis of random fluctuations (Theiler et al., 1992).

## Materials and Methods

### Population

Heart rate variability series were originally collected to estimate heart rate off-kinetics to 60-m sprint run (Storniolo et al., 2017) and re-processed to link heart rate off-kinetics parameters to frequency domain pre-exercise and post-exercise autonomic function markers (Storniolo et al., 2020). In this study the same data were re-analyzed by symbolic analysis with special emphasis on the PLATEAU phase. Although described in detail elsewhere (Storniolo et al., 2017), a summary of the original protocol and data acquisition procedure is provided in the following. The database comprised 21 subjects (15 men and 6 women, age: 24.9 ± 5.1 years, height: 178.4 ± 7.6 cm; weight: 72.1 ± 8.9 kg; mean ± standard deviation). Subjects were not suffering for any cardiovascular disease and any autonomic control impairment. The peak of oxygen consumption was 42.2 ± 8.7 ml⋅kg^–1^⋅min^–1^ as determined via an incremental running test performed on a treadmill on a different day. Subjects were physically active, being involved in either recreational activity or amateur sport activity with a maximum of three sessions per week. Each training session lasted less than 2 h.

### Experimental Protocol

Subjects performed a 60-m maximal sprint accomplished on an outdoor athletic track. The subjects were instructed to arrive at the experimental session in a relaxed and fully hydrated state. Additionally, they were told to avoid alcohol in the previous 24 h and caffeine in the previous 6 h before the test. Subjects were asked to perform the sprint at their best.

### Data Acquisition

The 60-m maximal sprint exercise was preceded by a short warm-up (5 min with jogging and stretching) and 10 min of resting period featuring 5 min in a seated position and 5 min in a standing position. Standing always followed seating. RR variability series was recorded using a heart rate monitor with transmitter belt (Polar S410, Kempele, Finland) during the resting, running and recovery periods. The standing phase just before starting the sprint was taken as pre-exercise condition.

### Selection of the Beat-to-Beat RR Variability Series

The end of the exercise was a recorded event by the smart watch. The onset and the offset of the PLATEAU phase were defined, respectively, as the first and the last pair of consecutive RR values found after the end of exercise and featuring a variation smaller than 15% of the maximal heart rate. We checked that the difference between the first and the last RR of the PLATEAU sequence was less than 15% of the maximal heart rate as well. The duration of the PLATEAU was 21.9 ± 7.5 cardiac beats, corresponding to 8.93 ± 2.75 s, with a minimum and maximum duration of nine and 38 cardiac beats, respectively. The RR sequence following the PLATEAU phase was fitted by an exponential function to detect the full return to a stable RR mean and to define the starting point of the post-exercise period. All tests were performed in the morning (i.e., 10–11 a.m.) to limit the influence of circadian rhythm on muscle performance and HRV. The analyses in the pre-exercise and post-exercise phases were carried out over sequences of the same length as that of the PLATEAU. These RR sequences were labeled as PRE and POST sequences, respectively. Matching was made in the beat-to-beat domain. The PRE and POST sequences were randomly selected within the overall pre-exercise and post-exercise periods via an automatic procedure choosing by chance the onset and controlling that the corresponding offset remained within the considered phase. We checked that the percent difference between the first and the last RR of the selected PRE and POST sequences was less than 15%. If this condition was not fulfilled a new random selection within the overall pre-exercise and post-exercise periods was performed until it was possible to find PRE and POST sequences fulfilling this criterion. The selected PRE, PLATEAU and POST sequences were linearly detrended before performing spectral and symbolic analyses. The RR mean and variance were indicated as μ and σ^2^ and expressed in ms and ms^2^, respectively.

### Spectral Analysis of RR Series

Parametric power spectral analysis was performed over PRE, PLATEAU, and POST sequences. The series were described by an autoregressive (AR) model whose order was fixed to five. This is the minimal model order that has the possibility of detecting a component at 0 Hz and two components with central frequencies different from 0 Hz that can fall into the LF and HF bands. This low model order allowed the estimation of the coefficients of the AR model even over the shortest sequence. Power spectral density was estimated from the identified coefficients of the AR model and the variance of the prediction error (Task Force of the European Society of Cardiology the North American Society of Pacng Electrophysiology, 1996; Porta et al., 2007b). The power spectral density was decomposed into spectral components. Each component was classified as LF or HF according to their central frequency (Task Force of the European Society of Cardiology the North American Society of Pacng Electrophysiology, 1996; Porta et al., 2007b). The sum of the power of all HF components was expressed in absolute units (i.e., ms^2^) and termed HFa. HFa was taken as a marker of vagal modulation (Akselrod et al., 1981; Pomeranz et al., 1985). The sum of the power of all LF components expressed in absolute units was calculated as well. The ratio of the LF power to the HF one (LF/HF) was taken as a marker of the state of the sympathovagal balance (Task Force of the European Society of Cardiology the North American Society of Pacng Electrophysiology, 1996; Pagani et al., 1997).

### Symbolic Analysis of RR Series

Symbolic analysis has been detailed elsewhere (Porta et al., 2001). Briefly, a uniform quantization procedure over ξ symbols was applied. The full range of the RR dynamics, namely the difference between the RR maximum (RR_max_) and the RR minimum (RR_min_), was divided into ξ bins of amplitude (RR_max_-RR_min_)/ξ and any RR value falling into a given bin was substituted with a symbol ranging between 0 and ξ-1. This procedure turned the RR sequence into a series of symbols. From the symbolic series we created patterns formed by *L* consecutive symbols. Traditional setting, namely ξ = 6 and *L* = 3, was exploited (Porta et al., 2001, 2007b). Given this setting, patterns could be classified according to the significance and sign of the variations computed over two consecutive symbols. Four families of patterns could be identified: stable patterns with no variation (0V) exhibiting identical symbols; patterns with one variation (1V) featuring two consecutive equal symbols while the remaining one was different; patterns with two like variations (2LV) presenting symbols that were all different but the sign of the two variations was equal; patterns with two unlike variations (2UV) featuring variations between two consecutive symbols different from zero and having opposite sign. Detected patterns belonged to one and only one of these four families. The results of symbolic analysis were expressed as percentages of patterns belonging to a given family over the total number of patterns. It was demonstrated that the 0V% increases in situations of augmented sympathetic control and vagal withdrawal, while the 2UV% raises in experimental conditions provoking vagal control activation and sympathetic withdrawal (Porta et al., 2001, 2007a,b; Guzzetti et al., 2005).

### Simulations

We tested the ability of spectral and symbolic analyses carried out over very short data sequences to distinguish two AR processes with power spectral density typical of RR series. We created 20 realizations of AR processes with central frequencies at 0.1 and 0.25 cycles/beat, respectively and pole modulus equal to 0.9. The realizations were rescaled to have mean and variance typically found during PLATEAU period (i.e., 400 ms and 10 ms^2^, respectively). Two types of simulations were considered (Tobaldini et al., 2009): (i) an AR process with the LF component whose variance was two times the one of the HF one, termed as AR_LF_, simulating RR series with dominant slow oscillations; (ii) a process with the opposite balance between the variance of the LF and HF components, termed as AR_HF_, simulating RR series with dominant fast oscillations. Spectral and symbolic analyses were carried out using a frame length of 10, 20, 30, and 40 samples, namely the typical length of the sequences analyzed in this study. We tested the ability of the two methods to differentiate AR_LF_ and AR_HF_ realizations assigned a given frame length by comparing the same index computed over the AR_LF_ and AR_HF_ realizations.

### Testing the Null Hypothesis of Random RR Dynamics

To exclude that the PRE, PLATEAU, and POST dynamics were fully random, a surrogate data approach was utilized. Surrogate series were generated by randomly shuffling the temporal order of the samples (Theiler et al., 1992). These surrogate series preserved the same distribution as the original series but destroyed any temporal pattern (Theiler et al., 1992). We generated one surrogate series for each RR dynamic. Spectral and symbolic markers were computed over original and surrogate series. If the markers computed over original data were significantly different from those assessed over surrogate series, the null hypothesis of random dynamics was rejected and we concluded that the series exhibited a certain degree of determinism.

### Statistical Analysis

Statistical analysis was carried out over original values of the markers. Two-way repeated measures analysis of variance (two factor repetition, Holm-Sidak test for multiple comparisons) was carried out. The two factors were length of the sequences (i.e., 10, 20, 30, and 40 samples) and type of dynamic (i.e., AR_LF_ and AR_HF_) in simulated data and period of analysis (i.e., PRE, PLATEAU, and POST) and type of data (i.e., original or surrogate series) in experimental data. Exclusively over the LF/HF ratio, two-way analysis of variance (Holm-Sidak test for multiple comparisons) was performed given that this marker could not be computed over all subjects in all periods of analysis and in both types of series. Statistical analysis was carried out using a commercial software (Sigmaplot, Systat Software, Inc., Chicago, IL, United States, version 11.0). Results are expressed as mean ± standard deviation. A *p* < 0.05 was always considered significant.

## Results

### Methodological Results Over Simulated Series

**FIGURE 1:**
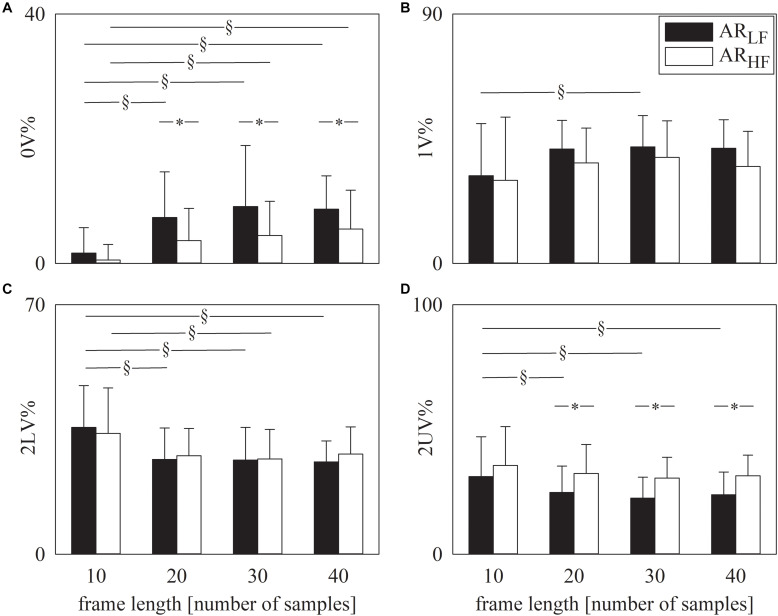
The grouped error bar graphs show the symbolic markers, namely 0V% **(A)**. 1V% **(B)**, 2LV% **(C)**, and 2UV% **(D)** computed over the simulated series as a function of the frame length (i.e., 10, 20, 30, and 40 samples). Indexes were calculated over AR_LF_ (solid black bars) and AR_HF_ (solid white bars) series. Data are reported as mean plus standard deviation. The symbol § indicates *p* < 0.05 across analyses carried out with different frame lengths within the same type of signal (i.e., AR_LF_ or AR_HF_ series). The symbol * indicates *p* < 0.05 between types of series analyzed with the same frame length.

**FIGURE 2:**
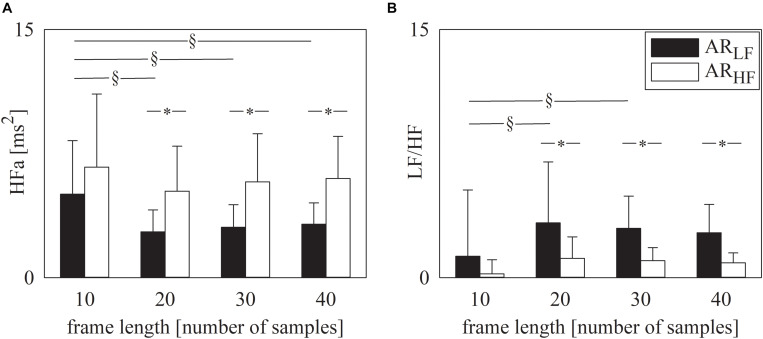
The grouped error bar graphs show the spectral markers, namely HFa **(A)** and LF/HF ratio **(B)** computed over the simulated series as a function of the frame length (i.e., 10, 20, 30, and 40 samples). Indexes were calculated over AR_LF_ (solid black bars) and AR_HF_ (solid white bars) series. Data are reported as mean plus standard deviation. The symbol § indicates *p* < 0.05 across analyses carried out with different frame lengths within the same type of signal (i.e., AR_LF_ or AR_HF_ series). The symbol * indicates *p* < 0.05 between types of series analyzed with the same frame length.

The grouped error bar graphs of Figure 1 show the results of symbolic analysis over simulated AR series. Symbolic markers, namely 0V% (Figure 1A), 1V% (Figure 1B), 2LV% (Figure 1C), and 2UV% (Figure 1D) are given as a function of the frame length set in the analysis (i.e., 10, 20, 30, and 40 samples). Symbolic indexes were computed over AR_LF_ (solid black bars) and AR_HF_ (solid white bars) series. Provided that the frame length was longer than 10 samples, 0V% (Figure 1A) and 2UV% (Figure 1D) were different over AR_LF_ and AR_HF_ series. As expected, 0V% was larger and 2UV% was smaller in AR_LF_ than in AR_HF_ series. The indexes 1V% (Figure 1B) and 2LV% (Figure 1C) were unable to distinguish AR_LF_ and AR_HF_ dynamics and this conclusion held regardless of the frame length. Remarkably, values of symbolic markers did not change across frame lengths within the same type of simulated series provided that the frame length was longer than 10 samples (i.e., 20, 30, or 40 samples) and this conclusion held regardless of the symbolic index (Figures 1A–D).

Figure 2 has the same structure as Figure 1 but it shows the results of spectral analysis, namely HFa power (Figure 2A) and LF/HF ratio (Figure 2B). Results are in line with those shown in Figure 1. Indeed, provided that spectral analysis was carried out over sequences longer than 10 samples, both HFa (Figure 2A) and LF/HF (Figure 2B) indexes could indicate that the AR_LF_ sequences had a dominant LF component, while the AR_HF_ featured a more important HF component. Similarly to the symbolic analysis, assigned the type of simulated series, results were similar across different frame lengths provided that they were longer than 10 samples (Figures 2A,B).

### Experimental Results Over RR Sequences

**FIGURE 3:**
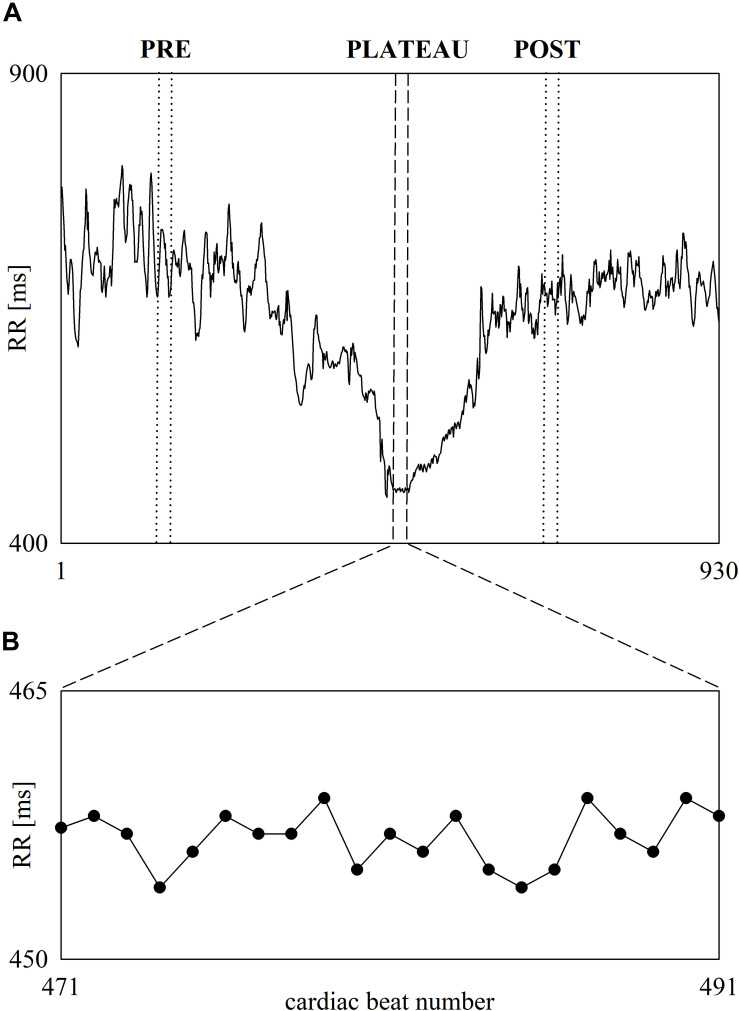
The line plot **(A)** shows an example of beat-to-beat RR dynamic recorded in one subject before, during and after 60-m maximal sprint. RR oscillates about a stable mean during standing just before the onset of the sprint. Then, the RR progressively shortens during the sprint and stabilizes during PLATEAU. Finally, RR starts lengthening and, then, oscillates about a stable mean value when the recovery is concluded. The vertical short-dashed lines denote the onset and the offset of the PLATEAU. The vertical dotted lines denote the onset and the offset of the selected sequences during PRE and POST phases of the same length as the PLATEAU. The inset shows the RR variability during PLATEAU **(B)**.

Figure 3 shows the beat-to-beat RR series recorded in a subject before, during and after 60-m maximal sprint test (Figure 3A). The first part of RR series is relevant to the pre-exercise phase (i.e., the resting phase while standing), while the last part to the post-exercise phase. In the middle the fast decrease of RR denoted the exercise phase. At the end of the sprint the RR remain stable for 20 cardiac beats and this period is labeled as PLATEAU. The onset and the offset of the PLATEAU are highlighted with two vertical short-dashed lines. Two vertical dotted lines mark the limits of the selected PRE and POST sequences of the same length, when expressed in number of RR values, as the PLATEAU. The RR dynamic during PLATEAU is magnified in Figure 3B. Despite the smallness of RR changes during PLATEAU, RR variations are clearly visible. Visual inspection suggested that these fluctuations are not compatible with random RR variations due to noise and, conversely, some determinism is present.

**FIGURE 4:**
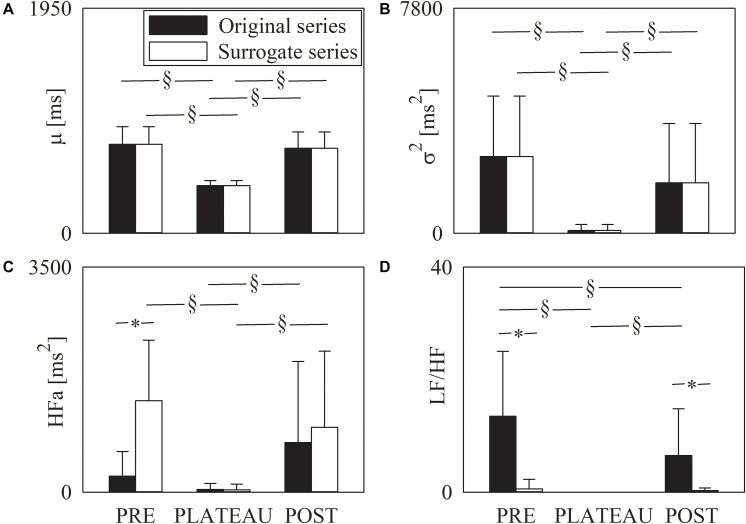
The grouped error bar graphs show time and frequency domain markers, namely μ **(A)**, σ^2^ **(B)**, HFa **(C)**, and LF/HF **(D)** computed over the RR series as a function of the period of analysis (i.e., PRE, PLATEAU, and POST). Indexes were calculated over original (solid black bars) and surrogate data (solid white bars). Data are reported as mean plus standard deviation. The symbol § indicates *p* < 0.05 across periods of analysis within the same type of series (i.e., original or surrogate sequence). The symbol * indicates *p* < 0.05 between types of series within the same period of analysis.

The grouped error bar graphs of Figure 4 show the time and frequency domain markers monitored in this study, namely μ (Figure 4A), σ^2^ (Figure 4B), HFa (Figure 4C), and LF/HF (Figure 4D) as a function of the period of analysis (i.e., PRE, PLATEAU, and POST). Time and frequency domain markers were computed over original (solid black bars) and surrogate (solid white bars) series. The μ significantly decreased during PLATEAU, both compared to PRE and POST. However, μ was not able to differentiate PRE and POST (Figure 4A). The σ^2^ exhibited similar trends (Figure 4B). By construction μ and σ^2^ computed over the surrogates replicated those of original data (Figures 4A,B). When computed over original data the HFa power increased during POST compared to PLATEAU, while no difference was observed between PRE and either PLATEAU or POST (Figure 4C). When computed over the surrogate data the HFa power was higher in PRE and POST compared to PLATEAU, while the HFa powers in PRE and POST were similar (Figure 4C). The HFa power of PRE sequences computed over surrogates was significantly higher than that over original data, while the HFa powers calculated over original data and surrogates were indistinguishable during PLATEAU and in POST (Figure 4C). The LF/HF ratio was 0 during PLATEAU because the LF component was never detected over both original and surrogate series (Figure 4D). As a consequence, over original data the LF/HF ratio was larger in both PRE and POST than during PLATEAU (Figure 4D). Moreover, over original series the LF/HF index was smaller in POST than PRE (Figure 4D). Over the surrogates the LF/HF ratio was similar across the experimental conditions with values smaller than 1 (Figure 4D). The LF/HF index computed over original series were significantly larger than that calculated over surrogates in PRE and POST (Figure 4D).

**FIGURE 5:**
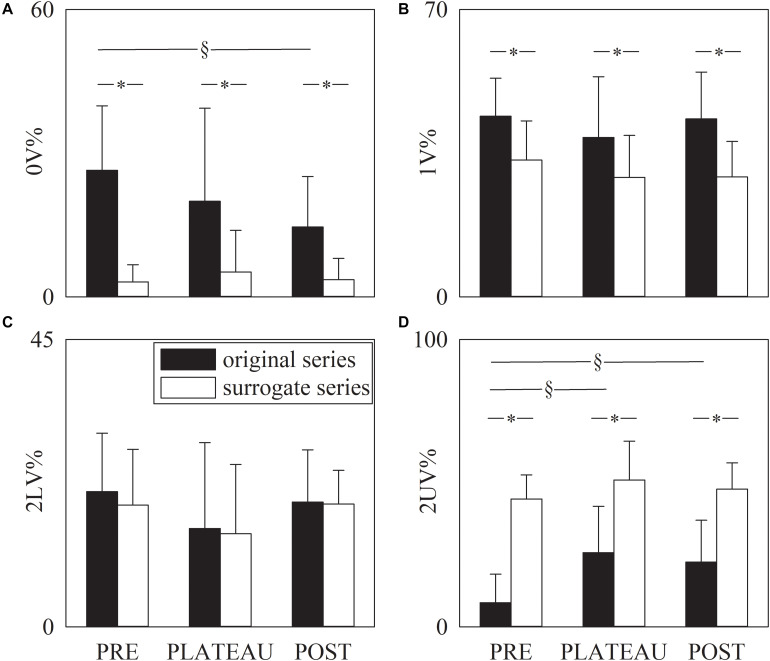
The grouped error bar graphs show the symbolic markers, namely 0V% **(A)**. 1V% **(B)**, 2LV% **(C)**, and 2UV% **(D)** computed over the RR series as a function of the period of analysis (i.e., PRE, PLATEAU, and POST). Indexes were calculated over original (solid black bars) and surrogate data (solid white bars). Data are reported as mean plus standard deviation. The symbol § indicates *p* < 0.05 across periods of analysis within the same type of series (i.e., original or surrogate sequence). The symbol * indicates *p* < 0.05 between types of series within the same period of analysis.

Figure 5 has the same structure as Figure 4 but it shows the symbolic indexes, namely 0V% (Figure 5A), 1V% (Figure 5B), 2LV% (Figure 5C), and 2UV% (Figure 5D). Over original data the 0V% significantly decreased in POST compared to PRE, but 0V% was not able to distinguish PLATEAU from PRE and POST (Figure 5A). Over the surrogate series 0V% did not vary with the period of analysis (Figure 5A). The 1V and 2LV% were similar in PRE, PLATEAU and POST and this result held for both original and surrogate series (Figures 5B,C). Over original series the 2UV% increased during PLATEAU and in POST compared to PRE (Figure 5D) but no significant difference was detected between PLATEAU and POST (Figure 5D). Over the surrogate series the 2UV% was stable in PRE, PLATEAU and POST (Figure 5D). The 0V, 1V, and 2UV% was significantly smaller in surrogates than in original series and this finding held regardless of the period of analysis (Figures 5A,B,D). Conversely, no separation between original and surrogate data was achieved using 2LV% in all the periods of analysis (Figure 5C).

## Discussion

The main findings of this study can be summarized as follows: (i) over simulated series both symbolic and low order spectral analyses were found to be able to differentiate dynamics with different spectral profiles when the analysis was carried out over sequences longer than 10 samples; (ii) over real RR series only symbolic markers were able to detect the underlying determinism of the cardiac autonomic control during PLATEAU; (iii) during PLATEAU symbolic analysis detected a sizable vagal control in presence of a significant tachycardia and a remarkably low respiratory sinus arrhythmia; and (iv) symbolic analysis carried out in POST sequences suggested that vagal control was more active and sympathetic regulation was more limited than in PRE sequences.

### Symbolic and Low Order Spectral Approaches Are Suitable for the Analysis of Very Short Sequences Taken From Simulated Series

Simulations proved that both spectral and symbolic analyses exhibited significant performances in separating different dynamics with values that remained stable across different frame lengths in the range from 20 to 40 samples. This finding assures the exploitation of both approaches in the present context. The remarkable performance of symbolic analysis cannot be taken for granted given that parameter setting, namely ξ = 6 and *L* = 3, was optimized over sequences of about 250 samples (Porta et al., 2001, 2007b). Results of simulations suggested that, although most of the words cannot be identified due to the shortness of the data sequences, the ability of separating different dynamics is still preserved. This good performance is likely due to the ability of the strategy grouping patterns into a very small number of families (i.e., four classes), thus providing the possibility that, although the majority of the words could not be identified, pattern classes could. The good score of spectral analysis might be a consequence of simulating AR processes. This situation might have favored our spectral analysis approach that models the series as an AR process (Baselli et al., 1997). The choice of identifying exactly the number of coefficients of the generation model (i.e., 5) might have produced an additional advantage. Given the remarkable performance of symbolic analysis over very short sequences, it should be preferable to spectral approach in contexts in which the mechanism of generation of the dynamic is unknown as it happens in real world.

### Symbolic Analysis Is More Powerful Than Spectral One Over Very Short Sequences Taken From Real RR Series

The difficulties of spectral analysis over real, very short, RR sequences took the form of the missing ability in differentiating original and surrogate data during PLATEAU phase. This disappointing result is the consequence of the irrelevance of the RR changes and impossibility of detecting LF components during PLATEAU. Conversely, the null hypothesis of random fluctuations was rejected by 0V, 1V, and 2UV% markers in all the periods of analysis. In particular, the rejection of the null hypothesis of random RR changes during PLATEAU suggested that cardiac autonomic control is operating during this phase and its deterministic action can be described by symbolic indexes despite the very small amplitude of RR fluctuations.

### During PLATEAU Tachycardia and RR Variability Reduction Mask a Sizable Vagal Modulation

This study is focused on the PLATEAU phase after maximal sprint exercise (Storniolo et al., 2020). This phase is usually poorly analyzed given that it is discarded by modeling approaches using exponential fitting (Imai et al., 1994; Pierpont et al., 2000; Pierpont and Voth, 2004; Buchheit et al., 2007; Peçanha et al., 2014) and it is underscored by more traditional analyses of heart rate recovery after maximal exercise in athletes exploiting a time resolution of minutes (Crisafulli et al., 2004; Sainas et al., 2016). At difference with the present study the analyses of post-exercise RR variability present in literature targeted the rapid RR modifications starting just after the PLATEAU phase. These analyses range from the simple assessment of the RR lengthening observed at a given time after the cessation of the exercise (Savin et al., 1982; Cole et al., 1999; Nishime et al., 2000; Kannankeril et al., 2004; Watson et al., 2017) to modeling approaches assessing the time constants of the exponential RR lengthening and the residual about the fitting (Imai et al., 1994; Pierpont et al., 2000; Pierpont and Voth, 2004; Buchheit et al., 2007; Peçanha et al., 2014) and to more sophisticated HRV analysis requiring time-varying or short-time spectral approaches (Arai et al., 1989; Kaikkonen et al., 2007; Martinmäki and Rusko, 2008). These approaches are sometimes applied in the presence of pharmacological interventions blocking alternatively one of the two arms of the autonomic nervous system directed to the sinus node or both branches (Savin et al., 1982; Imai et al., 1994; Kannankeril et al., 2004). We stress that in the present study the conclusion was drawn in physiological conditions, from very short, stationary RR sequences during PLATEAU and using a model-free method that does not impose *a priori* either the class of the underlying trend (e.g., exponential function) or the complexity of the model (e.g., mono- or bi-exponential).

During PLATEAU we observed signs of a dominant sympathetic control such as a significant tachycardia, a dramatic reduction of the magnitude of the RR changes (i.e., σ^2^) and respiratory sinus arrhythmia (i.e., HFa power) compared to PRE sequences. Despite the small magnitude of the RR changes, the percentage of highly variable RR patterns (i.e., 2UV%) significantly raised during PLATEAU compared to PRE sequences, thus suggesting a remarkable vagal modulation. The dependence of σ^2^ and HFa power on μ (Boyett et al., 2019), affecting mainly the HRV markers expressed in absolute units (Zaza and Lombardi, 2001; La Rovere et al., 2020), might have prevented the full comprehension of the relevance of the vagal control during PLATEAU, thus stressing the limits of time and frequency domain markers in this context. Conversely, since symbolic markers are normalized by definition between 0 and 100, they are not sensible to this bias (Zaza and Lombardi, 2001; La Rovere et al., 2020). This result suggests that an active vagal regulation occurs just before that the RR lengthening becomes visible. This vagal control might be even a residual parasympathetic modulation present during exercise (Kannankeril and Goldberger, 2002). The unvaried 0V% during PLATEAU compared to PRE sequences pointed to the persistency of the sympathetic control. Remarkably, this conclusion cannot be achieved from the LF/HF marker due to the missed possibility of estimating the LF power. These considerations are more in agreement with an early reactivation of the vagal control and a late sympathetic withdrawal (Arai et al., 1989; Imai et al., 1994; Cole et al., 1999; Nishime et al., 2000; Kannankeril et al., 2004; Pierpont and Voth, 2004; Coote, 2010) more than with a sympathetic withdrawal starting immediately after the end of the exercise and a late vagal rebound (Savin et al., 1982). The persistency of a high sympathetic control in the early recovery phase during PLATEAU in presence of a reactivation of the vagal control is consistent with the interpretation reported in Pierpont et al. (2000) suggesting that after a maximal exercise sympathetic control might alter the typical time constant describing vagal reactivation (Borresen and Lambert, 2008).

### POST Symbolic Markers Are Significantly Different From PRE Symbolic Indexes

In our experimental protocol the POST sequences were taken during the post-exercise phase after the full return to a stable RR mean. The fastness of the RR recovery is at difference with several studies that reported a slow increase of the RR mean over minutes (Savin et al., 1982) or even longer periods (Hautala et al., 2001; Javorka et al., 2002; Murrell et al., 2007). The full return to the baseline RR values is typical of this experimental protocol using a bout of maximal exercise lasting a few seconds given that different types of exercise might lead to different behaviors or need a longer period to attain pre-exercise states (Michael et al., 2017). Remarkably, 0V% significantly decreased in POST compared to PRE sequences, thus suggesting a less active sympathetic control in POST than PRE. This interpretation is corroborated by the decrease of the LF/HF ratio in POST compared to PRE. The trend of 0V% was associated to modifications of 2UV%. Indeed, the 2UV% was significantly higher in POST than PRE and this finding is compatible with a more active vagal control. Remarkably, changes of HRV markers occurred in absence of modifications of μ, thus supporting their full link with variations of the state of the autonomic nervous system (Zaza and Lombardi, 2001; Bauer et al., 2017; Boyett et al., 2019; La Rovere et al., 2020). Modifications of the autonomic control depend on type, duration, and intensity of exercise (Stanley et al., 2013; Michael et al., 2017). Usually, following an exercise of high intensity and long duration the magnitude of RR fluctuations decreased, thus suggesting a more limited vagal regulation compared to pre-exercise situation (Arai et al., 1989; Brown and Brown, 2007; Murrell et al., 2007). However, a less pronounced increase of the indexes of sympathetic modulation in response to active standing has been noted 90 min after a marathon (Murrell et al., 2007). A less active sympathetic control in presence of an orthostatic stimulus is compatible with the PRE-POST variation of 0V% observed in the present study given that both PRE and POST recordings are taken during standing (i.e., a lower sympathetic modulation is needed to cope with standing after exercise). It is worth noting that, even following a prolonged maximal exercise, trends of HRV markers compatible with a post-exercise increase of vagal control compared to PRE were observed provided that the time horizon of the monitoring period was suitably extended (Somers et al., 1991; Hautala et al., 2001).

## Conclusion

The study suggests that symbolic analysis can be fruitfully utilized to characterize the spontaneous RR fluctuations about the stable small mean observable for a few seconds following maximal sprint exercise. Results indicate that during PLATEAU a sustained sympathetic modulation is still present along with an early, and sizable, vagal reactivation. The vagal control rebound is completed at the end of the recovery period reaching a level higher than that observed before the sprint in presence of a similar mean heart rate. Given the peculiar state of the sympathovagal balance during PLATEAU, its characterization using symbolic analysis might open new possibilities of investigating sympathovagal interactions following maximal exercise and situations of potential imbalance between the two branches of the autonomic nervous system. Future studies should test the eventual link of the symbolic parameters during PLATEAU with the athlete’s performance and the possibility of modifying RR dynamics during PLATEAU using pharmacological challenges to fully validate their autonomic origin.

## Data Availability Statement

The raw data supporting the conclusions of this article will be made available by the authors, without undue reservation.

## Ethics Statement

The studies involving human participants were reviewed and approved by Ethical Review Board of the University of Milan. The data were reprocessed from Storniolo et al. (2017). The patients/participants provided their written informed consent to participate in this study.

## Author Contributions

AP and PC conceived and designed the study. JS performed the experiments. JS and BC analyzed the data. JS, BC, and AP prepared the figures and drafted the manuscript. All authors interpreted the results, edited and revised the manuscript, and approved the final version of the manuscript.

## Conflict of Interest

The authors declare that the research was conducted in the absence of any commercial or financial relationships that could be construed as a potential conflict of interest.
